# Warfarin Poisoning and Blunt Abdominal Trauma: A Rare Cause of Small Bowel Obstruction

**DOI:** 10.7759/cureus.13603

**Published:** 2021-02-28

**Authors:** Jane H Yeo, Claire Terez, Michael Shapiro, Aziz Merchant

**Affiliations:** 1 General Surgery, Rutgers New Jersey Medical School, Newark, USA; 2 Surgery, Rutgers New Jersey Medical School, Newark, USA

**Keywords:** small bowel obstruction, warfarin, hematoma, coagulopathy, sbo, intestine, trauma

## Abstract

Intestinal intramural hematomas are a rare complication of blunt abdominal trauma in the setting of anticoagulation. A 52-year-old male presented to our surgical service with high-grade small bowel obstruction secondary to an extensive small bowel intramural hematoma requiring resection. The patient sustained a blunt abdominal assault several days earlier and workup revealed severe coagulopathy likely secondary to overexposure to a warfarin-based substance. Few cases have been reported on coagulopathic traumatic small bowel hematomas causing small bowel obstruction. Current literature suggests non-operative management can be used safely; however, operative intervention is warranted if there are signs of ischemia or perforation. This case highlights the importance of a high index of suspicion, thorough investigation, and prompt intervention to avoid significant morbidity in small bowel obstruction secondary to intramural traumatic hematoma.

## Introduction

Intestinal intramural hematomas are caused by rupture of the terminal artery that arises from the mesentery and penetrates into the muscular layer of the intestinal wall. Bleeding within this space causes dissection of the muscularis mucosa and muscular layers [1,2]. As the hematoma grows, it causes additional vessel disruption and extension [2]. The resulting edema and hemorrhage can lead to mucosal thickening and possibly obstruction [3].

Spontaneous small intestine intramural hematoma is a rare complication of anticoagulant use, reported to occur in about one case per 2500 patients on anticoagulant therapy per year [4]. Conversely, intramural hematomas secondary to blunt abdominal trauma are a more common, yet still rare, phenomenon [5,6]. We present the case of an anticoagulated patient with a small bowel obstruction secondary to an intramural hematoma from blunt abdominal trauma, requiring emergent bowel resection.

## Case presentation

The patient is a 52-year-old incarcerated male with a past medical history of diabetes mellitus, chronic pancreatitis, and remote history of open cholecystectomy who was transferred to the hospital for complaint of abdominal pain. He reported three days of worsening epigastric and periumbilical abdominal pain with associated nausea and obstipation. He also reported two days of hematuria without dysuria or frequency. Four days prior, he was awakened by another prisoner who was kicking him in the abdomen.

On presentation, he was tachycardic but normotensive. His abdomen was distended and diffusely tender. Initial lab work was notable for leukocytosis of 17 and significant coagulopathy with an unmeasurable international normalized ratio (INR), partial thromboplastin time (PTT) > 200, and prothrombin time (PT) > 120. Repeat labs confirmed the coagulopathy. He denied any ingestions or known exposures. He was given two units of fresh-frozen plasma (FFP) as well as vitamin K and his coagulopathy began to correct.

**Figure 1:**
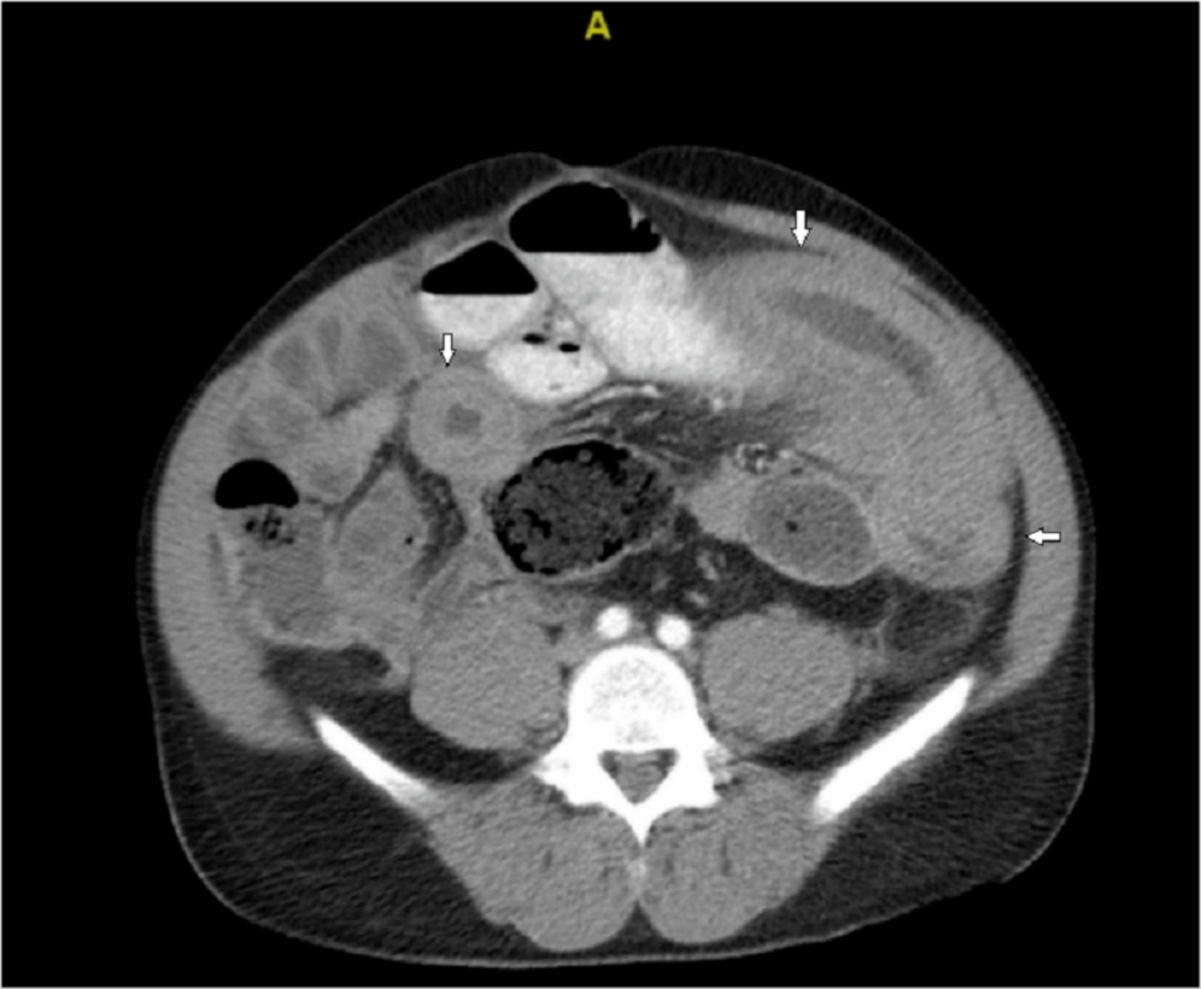
A CT scan of the abdomen revealing thickened small bowel

A CT scan of the abdomen/pelvis revealed significant bowel dilatation with mural edema and thickened loops of jejunum (Figure 1). There was fecalization of the proximal small bowel consistent with high-grade small bowel obstruction. Given his tachycardia, obstructive symptoms, diffuse abdominal pain and CT findings, there was concern for intestinal ischemia and the decision was made to perform an exploratory laparotomy. On inspection, the mid-small bowel was dilated with about 30 centimeters of thickened small bowel with circumferential hematoma. The entirety of the small bowel was examined and the point of obstruction appeared to be the extensive small bowel hematoma. This portion was resected and primarily anastomosed. The abdomen was closed and the patient was brought to the recovery room.

His post-operative labs were notable for a normalizing INR of 1.4 and PTT of 30. Given the unclear etiology for his extreme coagulopathy, Hematology was consulted. He had no family history of coagulopathies or hematologic disorders. He reported no history of bleeding symptoms prior to this episode. Coagulation studies were consistent with a deficiency of vitamin K dependent clotting factors that corrected with mixing studies. Differential diagnosis included common pathway factor deficiency, inhibitor exposure, or false result. Disseminated intravascular coagulation (DIC) and liver failure were ruled out. As his coagulopathy quickly corrected with FFP and vitamin K, the most likely etiology was exposure to overdose of a warfarin-based substance. He received additional vitamin K and recovery was otherwise unremarkable. Hematuria had resolved with correction of the coagulopathy. He was discharged on the seventh post-operative day to the prison infirmary. The final pathology was notable for portion of small intestine with transmural hemorrhage with clear resection margins.

## Discussion

There have been numerous reports of spontaneous intramural hematomas secondary to anticoagulant use [7]. These complications most commonly occur with vitamin K antagonists such as warfarin [8]. However, they have also been seen with low molecular weight heparin, cirrhosis, malignancies, and hematologic disorders [9,10]. Presenting symptoms include abdominal pain, nausea, emesis, abdominal distension, hematemesis, and melena [10]. Rarely, intramural hematomas can result in small bowel obstruction. Diagnosis is usually made on imaging or exploration, with CT imaging being the study of choice [8,10]. Coagulopathic induced intramural hematomas tend to involve long segments of short bowel, commonly affecting the jejunum followed by ileum and duodenum [10]. Characteristic imaging findings include symmetric circumferential wall thickening, intramural hyper density, and luminal narrowing.

Due to the uncommon presentation, there is no evidence-based literature suggesting standardized treatment [11]. Most studies consist of case reports or series. Studies have reported effective non-operative management with nasogastric decompression, bowel rest, and correction of coagulopathy [8,12-14]. A review of the literature revealed two case studies wherein poisoning lead to coagulopathic small bowel hematomas resulting in obstruction. Each case was successfully managed non-operatively with decompression, bowel rest, and correction of coagulopathy with FFP and vitamin K [13-14]. However, small intestine hematomas can lead to significant morbidity and operative intervention is indicated when there is concern for intra-abdominal hemorrhage, ischemia, perforation, peritonitis, or failure of non-operative management [1,8,11].

Likewise, in the case of blunt abdominal trauma, bowel and mesenteric injuries are uncommon presentations, reportedly found in 3%-5% of patients [5]. Specifically, small bowel injuries are present in only about 1.7% of all blunt abdominal injuries in the National Trauma Database. More rarely, these injuries can cause small bowel obstruction, usually from obstructive adhesions, perforation, mesenteric defect, ischemia, or intramural hemorrhage [15]. Often, these symptoms develop weeks to months after the traumatic episode.

Intramural hemorrhage in blunt abdominal trauma often occurs in the duodenum [16,17]. This is thought to be due to its fixed position over the vertebral column and disruption of mesenteric attachments [2,3,16-17]. Jejunal mural hematomas have been reported, however, they are more commonly associated with coagulopathies or systemic disorders [6,9-10]. There is higher reported incidence of jejunal injuries in pediatric populations after abdominal wall trauma likely due to the decreased abdominal musculature [3,6]. On CT imaging, traumatic hematomas are usually characterized by short focal segment of the affected bowel compared to more extensive involvement seen in coagulopathic bleeding [10]. Case reports have shown resection of the affected segment afforded good outcomes for the majority of patients with complications arising from missed or delayed diagnosis [6,15].

This case highlights a rare case of both anticoagulant overexposure and blunt abdominal trauma leading to small bowel obstruction. It should be noted that the diagnosis of intestinal hematoma was not correctly identified on the preoperative CT scan. The extensive nature of his small bowel hematoma suggests that his hematoma occurred due to his extreme coagulopathy in conjunction with blunt abdominal assault. The length of small bowel involved is not common in traumatic injuries alone nor spontaneous bleeds [10]. The extensive nature of his small bowel hematoma suggests that his assault occurred in conjunction with the anticoagulant poisoning. The injuries sustained during the assault likely progressed over the next several days aided by his supratherapeutic state, resulting in his symptoms of increasing abdominal pain, nausea, and obstipation. Prompt operative intervention was warranted and he underwent resection with primary anastomosis without complications.

## Conclusions

We present a rare case of multifactorial small bowel hematoma causing obstruction. Spontaneous intramural hematoma on anticoagulation and blunt abdominal trauma are both rare etiologies for bowel obstruction. This case highlights the importance of a high index of suspicion, thorough investigation, and prompt intervention to avoid significant morbidity in small bowel obstruction.
